# Multimodal Treatment of Gastric Cancer: Surgery, Chemotherapy, Radiotherapy, and Timing

**DOI:** 10.1155/2012/246290

**Published:** 2012-08-09

**Authors:** Marco Bernini, Lapo Bencini

**Affiliations:** Surgical Oncology, Careggi University Hospital, PAD.12 3/b, Largo Giovanni Alessandro Brambilla 3, 50134 Florence, Italy

Surgery is no more alone in the scenario of gastric cancer treatment since many years. Although the encouraging results of adjuvant chemotherapy obtained in Japan with S-1 were not achieved in Western countries, a novel approach, after the results of MAGIC trial, published in 2006, has been introduced by means of perioperative, or just preoperative, chemotherapy in the West, with many trials ongoing. 

Radiotherapy has been used from many years until now in the USA, with good results, and is part of many schedules in Europe as well. Chemotherapy has been proposed even in an intraperitoneal setting with hyperthermia, after extensive resection and peritonectomy (HIPEC). 

Novel drugs, known as immunological, have also been introduced, following gastric cancer molecular genetics studies, which appears to be a new frontier of translational research, as for many other cancers, in order to tailor a specific treatment to each case.

Similar to that achieved with the management of colorectal and esophageal cancers, multimodal treatment is therefore mandatory, to date, for gastric cancer, but timing, type of therapies, and schedules seem to be a puzzle yet.

The present special issue tries to span the diverse and wide possibilities of gastric cancer treatments and topics, from endoscopic dissection to HIPEC, without foregoing molecular genetics aspects, which are all part of nowadays clinical practice of surgical oncologists. Prominent interest is, obviously, in results in terms of overall survival with different approaches as well as complete response to preoperative treatments. Nonetheless, the impact of multimodal treatments on surgical perioperative complications and side effects should be taken into account, considering the aging of oncologic patients. 

One paper addresses a mini-invasive gastric cancer treatment, namely, submucosal dissection (ESD), which is a recent upgrade of the former mucosectomy. This technique is routine practice, when indicated, for early gastric cancers in Japan, while it is still not very well diffused in Western countries. The paper shows how this procedure is particularly helpful in elderly patients, without compromising the necessity of a subsequent gastrectomy, if needed.

Another paper shows the mechanisms of cisplatin-induced apoptosis and of cisplatin sensitivity. The authors also identify the molecular substrate called bridging integrator 1 (BIN1) to act as a potent predictor of cisplatin sensitivity in gastric cancer treatment. The importance of the translational research on clinical practice and patients selection for tailored chemotherapy is highlighted. 

One of the papers sheds light on an exquisitely surgical issue, which has been debated for so long: splenectomy. This was maybe, as admitted by the authors themselves, the most important bias of Dutch and British trials, making them favouring D1 because of higher surgical complications and mortality of D2. The paper encompasses very well the problem of splenectomy, which is not necessary to obtain a D2 lymphadenectomy and is detrimental in terms of complications without achieving longer survivals.

Another study focuses on the effects of neoadjuvant intraperitoneal/systemic chemotherapy (bidirectional chemotherapy) for the treatment of patients with peritoneal metastasis from gastric cancer. By the multivariate analysis many patients had some survival benefit, but the high morbidity and mortality require stringent patients selection.

The authors of another paper present the survival benefit of adjuvant radiation therapy for gastric cancer following gastrectomy and extended lymphadenectomy, demonstrating that the effect is not limited to patients treated with suboptimal surgery only.

The state of the art in the field of surgical treatment of peritoneal carcinomatosis from gastric cancer is reviewed by some authors. Concomitant systemic chemotherapy, cytoreductive surgery, and hyperthermic chemoperfusion showed promising results. Novel therapies, such as extensive intraperitoneal lavage and intraperitoneal targeted agents, are being applied in the management of this disease. 

Lastly a paper draws our attention to genetics of gastric cancer, from a pathogenesis point of view to the clinical use of some biomarkers. HER2 is the only universally detected and used to proceed with the biological therapy of Trastuzumab, while VEGF is a promising biomarker not only for prognosis but also for therapy as well. hERG1 is, instead, one of many other biomarkers that might play a role in gastric cancer clinical practice.

*Marco Bernini* *Marco Bernini* *Lapo Bencini* *Lapo Bencini*

